# Oral Dysbiosis and Neurodegenerative Diseases: Correlations and Potential Causations

**DOI:** 10.3390/microorganisms10071326

**Published:** 2022-06-30

**Authors:** Justine S. Nicholson, Kyle S. Landry

**Affiliations:** 1 Delavie Sciences, Worcester, MA 01605, USA; jsn2143@columbia.edu; 2 Department of Neurobiology, Columbia University, New York, NY 10027, USA; 3 Department of Health Sciences, Boston University, Boston, MA 02215, USA

**Keywords:** biofilm, health, neurodegenerative diseases

## Abstract

Biofilms are a heterogenous complex community of vegetative cells and extracellular polymeric substances that can adhere to various surfaces and are responsible for a variety of chronic and acute diseases. The impact of bacterial biofilms on oral and intestinal health is well studied, but the correlation and causations of biofilms and neurodegenerative diseases are still in their infancy. However, the correlations between biofilms and diseases such as Alzheimer’s Disease, Multiple Sclerosis, and even Parkinson’s Disease are starting to demonstrate the role bacterial biofilms have in promoting and exasperating various illnesses. The review article provides insight into the role bacterial biofilms may have on the development and progression of various neurodegenerative diseases and hopefully shine a light on this very important area of research.

## 1. Introduction

A biofilm is a community of microorganisms joined together and growing on a surface, existing in conjunction with one another. Biofilms can be widely diverse in structure, physiology, and species composition—even different strains of the same species can have different structural components and molecular mechanisms [1]. Some can be formed by a single bacterial species, but most are actually formed by multiple species, which vary depending on the environment in which the biofilm thrives [2].

Despite their differences, all biofilms have an extracellular matrix surrounding each individual component microorganism, which is formed by the microorganisms themselves—this is how multicellular communities join together into a single biofilm [3]. The adjoining of multiple species via this matrix can give biofilms qualities that are not characteristic of their individual component species, such as increased protection from environmental threats [1,4]. For instance, the matrix formed by biofilm microorganisms can trap nutrients, making them readily available to organisms in the biofilm and giving the biofilm increased virulence. This, combined with biofilm species’ polymicrobial interactions, makes many biofilms resistant to traditional antibiotic treatments [4,5].

When considering biofilms in human health, some biofilms can be beneficial for humans, such as assisting in nutrient absorption and biodegradation and providing resistance to infections [1]. Biofilms that form dental plaque, for instance, are actually composed of microbes that antagonize the colonization of harmful microorganisms in the mouth [3]. However, a vast number of biofilms are a threat to human health, mostly credited to their antibiotic resistance, which has made biofilms widely contribute to increased mortality and morbidity rates [4]. There are biofilms that cause chronic infections on medical implanted devices [3], or others that worsen some diseases in their formation during various disease states, such as in the lungs for Cystic Fibrosis [6]. Biofilms are also prominent in skin infections, such as diabetic foot ulcers, and can also cause chronic oral diseases such as dental caries and periodontitis [1,7]. The difficulty in eliminating these disease-causing biofilms makes treatment particularly difficult and can sometimes mean that drastic measures must be taken to prevent further damage or mortality—such as amputation for diabetic foot ulcer patients [7].

This review specifically focuses on oral biofilms, the oral microbiome, and how biofilms in the oral cavity can be associated with neurodegenerative diseases. The purpose of this review is to examine current findings that present these associations, discuss potential mechanisms that link oral biofilms and various human diseases together, and postulate whether this link between oral biofilms and neurodegenerative diseases is valid.

## 2. The Oral Microbiome, Oral Biofilms, and Oral Diseases

The moisture and warmth of the human oral cavity make it an optimal domain for several microorganisms. The variation in oral habitats—i.e., the tongue, teeth, cheeks, gums, tonsils, and hard and soft palates—also contributes to the extensive diversity of oral microbiota, as different microbes colonize different oral structures [8,9]. There are an estimated 500 to 700 distinct oral species in the mouth, including those that are uniquely found in individuals with oral diseases or in healthy patients, and many of which are difficult to cultivate and isolate outside of their natural oral habitat [10,11].

Common microbiota of the healthy oral cavity includes species of *Gemella*, *Granulicatella*, *Streptococcus*, and *Veillonella*, with the species *Streptococcus mitis* being the most commonly found in all oral cavity sites [9]. One of the reasons *Streptococci*, in particular, are among the most abundant in the oral cavity is because of their predominance in the oral biofilm, referred to as dental plaque, which forms on tooth surfaces [12]. This biofilm’s formation involves *Streptococci* adhering to salivary protein α-amylase that coats the surface of teeth [12]. While dental plaque is often thought of as detrimental, in actuality, it is extremely important for human oral health when the plaque microbiota are in correct balance [3,10]. The resident human oral microbiota of plaque biofilms act as a crucial barrier against external microorganism populations and thus act as a defense in favor of human oral health [10]. The relationship between humans and resident oral microbiota, therefore, is symbiotic.

While plaque biofilms are beneficial under regular circumstances, problems can occur when the crucial host-microbe balance is disrupted and when there is a substantial change to resident oral microorganisms’ habitat, either depleting the plaque biofilm—thus killing its microorganisms—or allowing the plaque to accumulate beyond acceptable levels for human oral health [10]. Any disruption of this kind can promote the overgrowth of previously minor components of the oral microbiome (such as oral yeasts) or the colonization of foreign, pathogenic microorganisms [10]. This disturbance is how oral infections and diseases can develop. For instance, studies have indicated that pathogens causing periodontitis, such as *Porphyromonas gingivalis*, can be found in the microbiota of a healthy human oral cavity but only in trace amounts. It is the buildup of oral plaque, thus causing the human body to have an inflammatory response, that promotes the overgrowth of these periodontal pathogen biofilms and causes significant infection [10].

The introduction of exogenous pathogens and/or overgrowth of previously existing resident oral microbiota in the mouth can cause not just periodontitis but a series of oral diseases and infections, which can involve harmful biofilms that colonize the oral cavity. Apart from periodontitis, these can include endodontic infections, which involve invasive growth of bacterial and fungal microbiota and dental caries, which are caused by the overgrowth of the predominant plaque species *Streptococcus mutans* [13,14,15].

Oral biofilms, oral diseases, and their associated microorganisms—such as periodontitis and its predominant biofilm-forming pathogen *Porphyromonas gingivalis*—have been shown to have strong associations with several systemic human diseases such as neurodegenerative diseases, cancers, diabetes, cardiovascular disease, and adverse pregnancy outcomes [9].

Outside of bacteria, numerous viruses, protozoa, fungi, and archaea can be found in the oral microbiome and can take up residence, even if temporarily, in an oral biofilm. Generally, the viruses that are found are transient; however, permanent, disease-associated viruses can be found in the oral microbiome. Hepatitis viruses, human immunodeficiency virus (HIV), *Herpes simplex*, *Herpes labialis*, and human papilloma virus (HPV) can be found in the oral microbiome during or after acute infection [16,17,18,19]. Protozoa and archaea are minor in terms of population, yet their potential impact on overall health is substantial. For example, the archaea methanogens *Methanobrevibacter oralis* and *Methanosarcina mazeii* are present in very low numbers in a healthy oral microbiome [20]. However, their numbers are significantly elevated in patients with periodontitis. This polymicrobial infection impacts 35% of US adults and can lead to significant health and dental complications [20,21]. Like archaea, protozoa are generally low in numbers in adults with good oral hygiene and are elevated in adults with poor oral hygiene and oral diseases [22]. The connection between health and the oral microbiome is not localized to the host. For example, there is a strong connection between a mother’s oral health and the bacterial transmission between mother and infant [23,24]. Extrinsic factors, such as cohabitation (human/human and human/animal), diet, environment, and genetic disposition, all impact the oral microbiome and its potential role in the overall health of the host [25,26,27,28].

## 3. Oral Biofilms and Neurodegenerative Diseases

Neurodegenerative diseases can be broadly defined as any disease which causes the human brain to deteriorate, usually leading to a loss in bodily function such as memory or motor movement. These diseases include Parkinson’s Disease (PD), Alzheimer’s Disease (AD), Multiple Sclerosis (MS), and Lewy Body Disease (LBD). There is a growing body of scientific evidence which associates oral dysbiosis—as in, microbial imbalances in the oral cavity—with various neurodegenerative diseases. Most research in this area focuses on Alzheimer’s Disease (AD), but more recent studies also relate oral dysbiosis with other neurological diseases, such as Multiple Sclerosis and Lewy Body Disease [29,30,31].

The central question of this review is: if there is a causal relationship between oral dysbiosis and various neurodegenerative diseases, what are the potential links? Does oral dysbiosis cause the neurodegenerative disease, is it the neurodegenerative disease that causes the oral dysbiosis, or is there even a connection involved?

### 3.1. Alzheimer’s Disease (AD)

Estimated to affect 4.5 million people in the U.S. and make up more than 20 million cases worldwide, Alzheimer’s Disease (AD) is now the most common neurodegenerative disease [32]. Its numbers are only expected to triple over the next few decades, making it one of the most heavily researched neurodegenerative diseases [33]. AD and other dementias are disorders of memory, typically starting with the loss of neurons in the internal cortex and around the hippocampus, although the disease can manifest in different areas. Certain neural regions may be more vulnerable to neuropathologic changes than others [34].

It is currently postulated that Alzheimer’s Disease (AD) is correlated with a buildup of abnormal and harmful proteins in the brain and nervous systems, such as amyloid-beta (Aβ) protein, the microtubule-associated protein tau, the lipid-carrier protein apolipoprotein E, and the presynaptic protein α-synuclein [35]. What makes these proteins harmful is that, due to their abnormal nature (often caused by mutations), they cannot be cleared from the brain with typical clearance mechanisms; they instead aggregate, forming senile plaques which are associated with oxidative stress and neuroinflammation which leads to neuronal death [35,36,37].

There is still debate regarding the diagnostic criteria for AD—formally speaking, it cannot be truly diagnosed until after death when the brain can be analyzed post-mortem for mutant protein aggregates. AD is most commonly characterized by the brain deposition of neurofibrillary tangles (caused by Tau) and Aβ plaques, and some researchers are of the view that either Aβ plaques or Tau fibrils are the cause of AD symptoms [37,38,39,40]. There are even some suggestions that neither Aβ plaques nor neurofibrillary tangles cause cognitive deficits in AD patients, but rather that they are products of changes in signaling pathways which result in mutations in the proteins of which they are composed [41,42]. For instance, it could be that errors in common signaling pathways can result in the appearance of mutant Aβ precursor protein or mutations of presenilins involved with cleaving Aβ precursor protein, which can physiologically affect synaptic function even before the appearance of plaques and tangles. Some AD cases are genetic, but most cases are sporadic and associated with environmental factors such as age, diet, sleep quality, and level of education [37], which may trigger genetic changes, thus causing mutations to encourage the formations of plaques and tangles. What has been explored more recently is how the environmental factor of oral health can contribute to AD. Because the mechanistic and environmental causes of AD are still widely unknown and debated, it is difficult to say how oral health and oral dysbiosis can be connected to this disease or if there is a direct relation at all.

It has been established that, generally, Alzheimer’s Disease can lead to oral dysbiosis—there is much evidence suggesting that Alzheimer’s patients naturally experience a decline in oral health after disease onset compared to healthy individuals [43]. By the disease’s nature, a decline in motor movement is observed, including a decline in mouth movement and in the limbs required to maintain oral health, such as brushing teeth. Therefore, the likelihood of developing caries and periodontitis in AD increases as a decline in oral health maintenance leaves more opportunities for the overgrowth of harmful microbes in the mouth [37].

**Figure 1:**
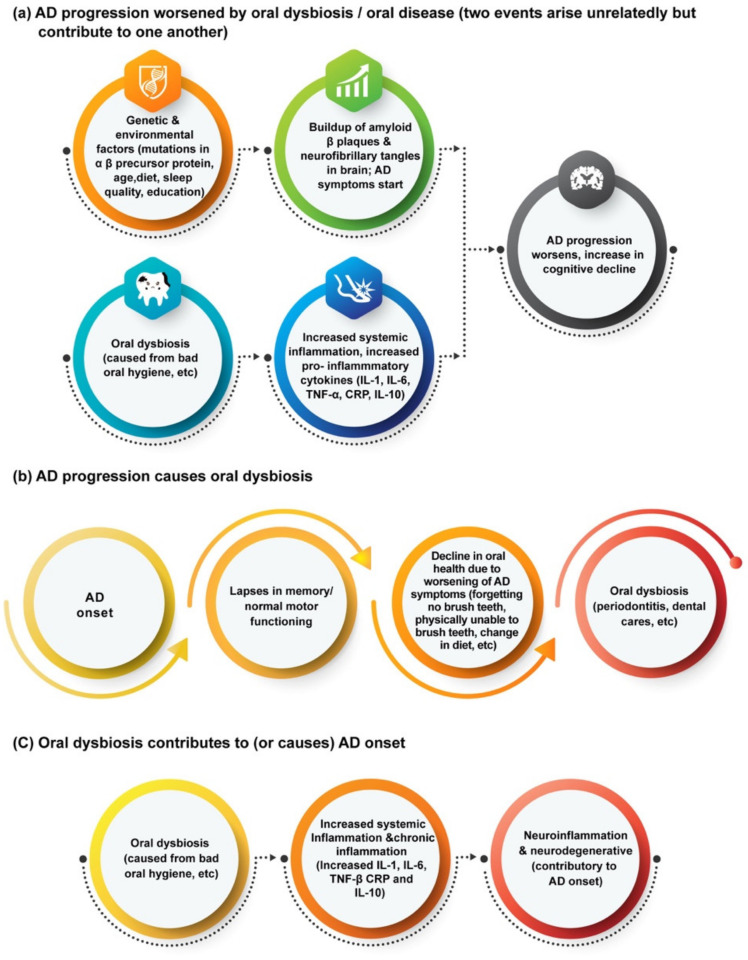
Potential links between oral dysbiosis and AD and other neurodegenerative diseases [35,36,37].

What is more complex is how these oral diseases can worsen, or even perhaps lead to, the onset of AD. The majority of reviews relating oral biofilms to neurodegenerative diseases focus on AD, particularly in its connection to periodontitis, which is an inflammatory oral disease caused by the dysbiosis of plaque biofilms leading to damage of the gums and structures supporting the teeth [44]. A currently popular theory is that the increased inflammation caused by periodontitis worsens the neuroinflammation involved with AD (Figure 1), as periodontal pathogens can release or induce pro-inflammatory cytokines such as IL-1, IL-6, and TNF-alpha [37,45]. For instance, Ide et al. conducted a cohort study that tracked the levels of pro-inflammatory markers C reactive protein (CRP), TNF-alpha, and IL-10 in the blood of 60 AD patients over a period of six months [46]. At the end of the study, they found that periodontitis at baseline was associated with increased levels of these markers and of the pro-inflammatory state over the six-month follow-up period, and Ide et al. concluded that periodontitis is associated with an increase in cognitive decline in AD, independent to baseline cognitive state [46]. This conclusion has also been supported by data from other studies [47].

It has also been suggested that AD is a direct consequence of bacterial invasion, with some studies postulating that oral microbiota—particularly periodontal pathogens—could be transported from the mouth into the brain via the bloodstream, leading to bacteremia [48]. How this would progress AD and the mechanism involved is still unknown.

However, the presence of periodontitis in AD patients could also be explained by confounding factors, such as a compromised or modified inflammatory or immune response which also leads to AD [46]. A study performed by Franciotti et al. (2021) quantified the abundance of *Porphyromonas gingivalis* (Pg), a common microorganism that causes periodontitis, in patients with neurodegenerative diseases (including AD), neurologic patients with no neurodegenerative disease, and healthy controls to determine a potential relationship between the neurodegenerative process and periodontitis severity. They found that the Pg abundance in the oral cavity of neurodegenerative diseased patients was significantly higher than that found in non-diseased and healthy groups, and from this, Franciotti et al. concluded that this evidence supported the hypothesis suggested by other studies that oral pathogens can induce a systemic antibody response, influencing the progression of the disease. They also speculated that this connection might be related to the efficacy of patients’ immune responses to periodontal disease [49]. It seems that only a correlation between periodontitis and AD and other neurodegenerative diseases has been established; the mechanism by which they are related is still unknown or has not yet been replicated in a controlled mouse study.

### 3.2. Parkinson’s Disease

Parkinson’s Disease (PD) is another common neurodegenerative disorder, affecting around one million people in the U.S. alone. As the age of the population increases, PD cases are also expected to increase since PD onset is more prevalent in older age groups [50,51].

Unlike Alzheimer’s Disease, which is a disease of memory, Parkinson’s is one of movement and vigor. It affects coordinated movement in fine and gross motor skills and is characterized by the loss of ability to voluntarily move one’s muscles (akinesia) and slow movement, and impaired ability to move one’s body swiftly on command (bradykinesia). However, increasing evidence suggests that bradykinesia presented in PD patients does not necessarily mean that they have lost the ability to move at faster speeds, but rather that the brain of these individuals with bradykinesia makes implicit, unconscious decisions to not move fast due to a shift in the cost/benefit ratio of energy expenditure necessary to move at a normal speed [52]. This is further supported by cases of ‘kinesia paradoxica’, or heroic lapses in PD, in which PD patients’ impaired movement momentarily disappears when they are in urgent situations—such as when an immobile patient leaps from their wheelchair to save a drowning man or help a child in danger [53].

Previously, at a neurochemical level, it was widely unknown what characterizes PD until it was discovered that the drug 1-Methyl-4-phenyl-l,2,3,6-tetrahydropyridine (MPTP), which has been used as a kind of heroin, can induce Parkinson’s symptoms. Since then, MPTP has opened new doors for Parkinson’s research, and its use has allowed for the creation of animal Parkinson’s models, as it produces parkinsonian symptoms and neuropathology [54]. MPTP is selectively toxic for dopaminergic cells, and so it is now widely known that PD is characterized by a lack of dopamine. Hence why, in Parkinson’s patients, L-DOPA is often used as a treatment because it is a precursor to dopamine [55]. Dopamine is a neuromodulator, and its release is specialized to specific neural areas, including the substantia nigra and the ventral tegmental area (VTA). Parkinson’s Disease primarily affects the substantia nigra, as PD patients are observed to have atrophy of midbrain dopaminergic neurons in this area. Since the substantia nigra is a crucial region involved with movement control, its atrophy alters the neural circuitry involved with movement initiation and movement vigor [56].

The pathology causing the death of dopaminergic neurons in PD is still widely unknown, but various factors have been proposed to play a role. It seems to result from alterations in mitochondrial function [56,57]. Alpha-synuclein pathology seen in Lewy-Body Disease (LBD) also appears to underlie PD. Alpha-synuclein can form Lewy-Bodies such as inclusions and can travel between neurons, extending PD pathology [58,59]. It has been shown that injecting alpha-synuclein fibrils into mouse brains can cause pathology to spread [60,61]. In a sense, similar to AD, most cases of PD are late onset (in later life) and do not have a clear inheritance pattern; however, there are a small fraction of PD cases that are genetic, such as in genetic mutations in the mitochondria of dopaminergic neurons such as PINK1 [62].

Similar to AD and other neurodegenerative diseases, the causes of non-inherited Parkinson’s Disease are widely unknown. Of course, exposure to dopaminergic neurotoxins such as MPTP and other herbicides and pesticides can lead to PD onset [56], but how PD arises naturally is still very much a mystery. Its nonhereditary causes have been suggested to be a multifactorial combination of genetic predisposition, environmental risk factors, and age-related processes [63]. Potential risk factors have been identified, such as dietary lipid and milk consumption, high caloric intake, and head trauma [64].

In relation to the potential links between PD and oral dysbiosis, there is very little research. There is correlative evidence that suggests a potential connection: for instance, PD patients have been found to have oral difficulties sometimes years before their diagnosis [65], and a higher frequency of poor oral health, caries, periodontal disease, and fewer remaining teeth have been reported in PD patients [65,66]. However, the causative mechanism that may be involved, if there is any, is still very unclear.

**Figure 2:**
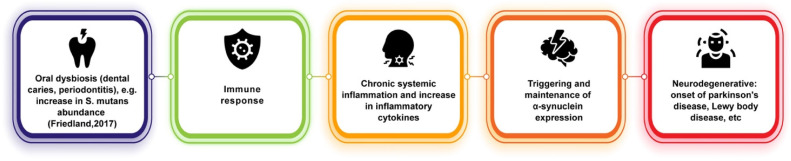
Potential link between oral dysbiosis and neurodegenerative diseases of alpha-synuclein pathology [72].

Similar to its connections with AD, oral dysbiosis and its contributions to systemic inflammation may play a role in PD onset (Figure 2). It has been hypothesized that the periphery of the body could be an early indicator of PD onset, as it has been observed that, as previously stated, phosphorylated alpha-synuclein aggregates can travel between neurons and are not only found in the central nervous system but also within the olfactory bulb and in the peripheral autonomic nervous system of the upper aerodigestive [67] and gastrointestinal tracts [68]. These can be traced very early during PD, even before the classic motor impairment symptoms emerge [65,69]. Other findings have suggested that phosphorylated-alpha-synuclein may underlie non-motor symptoms in PD, such as decreased salivary production and dysphagia [65,70]. Fleury et al. [71] showed that PD patients have different oral bacterial ecology than control patients and found that PD patients showed higher amounts of oral microbiota implicated in dental caries and periodontitis. In particular, the relative abundance of S. mutans was found to be increased in PD patients, which has been shown to be capable of amyloid formation and, in animal models, to potentially play a role in alpha-synuclein production and aggregation and cerebral inflammation [71,72]. Furthermore, pro-inflammatory cytokine levels were found to be increased in PD gingival crevicular fluid in this study. Much of this evidence is suggestive of underlying pathways involved in connecting oral dysbiosis and PD. Dysbiosis in PD could promote immune activation and systemic inflammation, thus upscaling pathogenic processes (such as triggering and maintaining alpha-synuclein expression) and establishing a chronic neuroinflammatory state [71].

Although a complex, multifactorial disease such as Parkinson’s is difficult to study in animal models, especially when considering its causes in relation to oral dysbiosis, there is a need for more animal models and controlled studies to manipulate the proposed molecular pathways involved in PD onset. It seems that only correlative studies have been proposed.

#### A Note on Lewy-Body Disease (Subsection of Parkinson’s Section)

Lewy-Body Disease (LBD), similar to PD, is another neurodegenerative disease that has alpha-synuclein pathology [60]. Because LBD is widely understudied and difficult to diagnose, the mechanisms by which it arises and whether or not oral dysbiosis contributes to this disease are overall unknown. However, due to some of its similarities with PD, it may be possible that similar mechanisms in PD are involved with LBD since there have been suggestions that oral dysbiosis may be involved with alpha-synuclein aggregation [71,72].

### 3.3. Multiple Sclerosis (MS)

With an estimated global prevalence of 35.9 per 100,000 people and 2.8 million people worldwide, Multiple Sclerosis (MS) is another common neurological disease that—unlike Parkinson’s and Alzheimer’s, which begins at a later age—typically presents itself around young adulthood [73]. It is a chronic inflammatory disease of the central nervous system, characterized by inflammatory demyelination and often caused by lesions in the brain or spinal cord. These lesions are a result of the immune system targeting myelin sheaths of neurons in the brain. This degeneration leads to neurological impairments over time, which increases disability, such as impaired motor movement in patients [30].

There are different forms of MS, but most patients fall into the relapse–remitting phase which consists of periods of normal function coupled with significantly worse experiences of disability and acute onset of new symptoms, usually triggered under certain conditions. In this stage, acute flares can be observed, but they tend to resolve back to normal functioning or slightly milder symptoms. The other form of MS, which can occur later in disease progression, is the secondary progressive phase in which relapses still occur, but they tend to die out as the disease progression worsens. This phase correlates with an increase in brain atrophy, and instead of many acute inflammatory relapses such as in the relapse–remitting phase, there is more of a chronic progression of demyelination and axonal loss [74].

The basic pathological process of MS consists first of the robust infiltration of T cells and macrophages into the brain. These cells attack the myelin sheaths of neurons, which leads to loss of axon conductance. This causes a weak signal of neurons and loss of various functions during MS relapse [75]. Especially during the relapse–remission phase of MS, in which more subtle axonal injury occurs during relapse periods, the relapses are somewhat reversible, and the injured axons can eventually recover [76]. This differs from other forms of injuries such as traumatic brain injury (TBI) or even the neurodegeneration in AD, in which, due to complete neuronal death, recovery from symptoms may not occur. In MS, with a loss of axon conductance, a weak signal with poor conductance during the period of relapse may occur, but there is potential for it to return to normal during remission periods.

MS has genetic contributions and susceptibilities, but these appear to be minor contributors. There have been polymorphisms observed in major histocompatibility complex, human leukocyte antigen (HLA) class II alleles for MS. HLA is responsible for presenting antigens to naive T Cells of the immune system, which suggests that the presentation of antigen to naive T cells is a key event in MS initiation. However, this genetic contribution is minimal, as some studies have found no evidence for interactions between classical HLA alleles and non-HLA risk-associated variants, and therefore there is likely a minimal effect of polygenic epistasis in modulating major risk alleles [77]. Furthermore, twin studies for MS have shown poor concordance (only about 30%), indicating that environmental factors play a more crucial role in MS onset [78]. It has been suggested that environmental factors such as low serum levels of vitamin D, smoking, childhood obesity, or viral infection may contribute to “triggering” the onset of the disease; however, these are just correlations [30].

In order to identify potential mechanistic etiologies of MS, it is important to understand the events and pathways leading to immune cells targeting myelin in the brain.

Current evidence suggests that CD4+ T cells of the immune system are crucial in the initiation and pathology of the disease [79]. Animal models have demonstrated that the disease can be adoptively transferred with CD4+ T cells, but the same cannot be performed with other immune cells such as CD8+ T cells or antibodies [80]. However, this may be specific to experimental autoimmune encephalomyelitis (EAE) animal models because human treatments which target MHC class II-restricted CD4+ T cells have not provided any clinical benefits, and they have not been more effective than treatments that target T cells and B cells more globally [81]. There seems to be nothing special about CD4+ T cell targeting in established diseases, but it may be important in disease initiation.

MS is widely considered to be an autoimmune disease, but it is under debate as to whether myelin proteins are the true immune stimulus. In other words, even if myelin is targeted, it may not be what the body is targeting. Therefore, the two leading hypotheses for how immune cells target neural myelin are that either (1) antigen-presenting cells (APCs) present myelin peptides to CD4+ T cells or (2) CD4+ T cells are activated by pathogenic antigens which resemble myelin peptides (via molecular mimicry) and drive specific immune responses which act against myelin peptides.

In support of the molecular mimicry hypothesis of MS, there has been a recent exploration into MS having a viral infection etiology. It has been shown that MS relapses often follow peripheral infections [82]. More recently, striking correlative evidence has pointed to the Epstein–Barr Virus (EBV) being the primary candidate for causing MS, suggesting that the presentation of EBV to APCs in the immune system mimics myelin peptides, therefore leading the immune system to “accidentally” target myelin peptides while trying to target the virus [83].

Although it is highly unlikely that oral dysbiosis plays a role in the MS initiation process in immune cells, there has been evidence in support of oral dysbiosis potentially contributing to worsening the disease’s progression via its roles in inflammation. Inflammation always plays a role in demyelinated lesions in MS, and when this inflammatory process is no longer active, neurodegeneration in MS patients is reduced to levels seen in control subjects [75]. Given that oral dysbiosis increases systemic inflammation, there may be common inflammatory pathways involved in causing acute relapses of symptoms in MS. In relation to oral microbiota and oral dysbiosis, there is very scarce research that has been performed for MS, such as PD [84,85].

There have been studies investigating the role of gut microbiota in shaping the relapse–remitting phases of MS [86,87]. Much like the suggested role of gut microbiota in Parkinson’s Disease pathogenesis, this may mean that oral microbiota may similarly play a role [65,87,88]. The mouth is the first entry point from the external environment for pathogenic microorganisms; it is, therefore, likely that gut microbiota is highly influenced by oral microbiota, and it is worth examining the impact of oral microbiota on systemic health more closely [65].

Like studies with other neurodegenerative disorders, in relation to oral dysbiosis, only correlations—and no causative mechanisms—have been noted between MS and oral dysbiosis. It has been postulated that peripheral inflammation caused by periodontitis (and its characteristic microorganisms) may also be involved with the etiology of MS, PD, and AD [65,89]. For instance, in addition to finding strong correlations of *Porphyromonas gingivalis* levels with AD, Franciotti et al. [90] also found a similar association with patients having MS, PD, and other neurodegenerative diseases, including frontotemporal dementia, dementia with Lewy Bodies, and Huntington’s Disease. This suggests that the inflammation caused by periodontitis may be a potential factor in worsening the progression of several neurodegenerative diseases.

It has also been suggested that oral microbiota can indirectly modulate the kynurenine pathway (KP). The KP has been implicated in tryptophan production, degrading neuroactive compounds, and producing kynurenine bioactive metabolites, which have immunologic and neurotoxic activities. Therefore, with the immune component, the KP may have functions in furthering MS progression. Immune kynurenines can suppress the immune response and could, therefore, further progress the disease [90].

## 4. Potential Remedies

Because the causative mechanism between oral biofilms and neurodegenerative diseases has not yet been established, it is difficult to identify remedies that break this connection. Furthermore, the etiology and pathophysiology of neurodegenerative diseases and treatments to better reduce their progression are still widely unknown. For now, however, given the potential links between the disease progressions and oral dysbiosis, it may be possible that restoring microbiota homeostasis may aid in reducing the progression—or, potentially, prevent the onset—of neurodegenerative diseases [91]. Currently proposed remedies for this involve the use of probiotics to restore levels of healthy bacteria and discourage levels of harmful pathogens [92], enzyme-related therapies [93], homeopathic therapy [94], or targeting the properties of specific microbial species using knowledge of the particular bacterial biofilm and the way it develops to limit or eradicate its growth [95]. More research can be performed in understanding the genetics of pathogenic biofilms and in developing a better understanding of how environmental signals are sensed and transduced in biofilm-forming bacteria, as well as the molecular mechanisms that biofilms utilize to respond to threats [14]

Overall, disease prevention has been emphasized in the literature, and very few treatments have been proposed following disease onset because there has not been true causation established. Therefore, the best current recommendations are to take actions that prevent the onset of oral diseases or limit risk factors for neurodegenerative disease (such as exercise, no smoking, maintenance of oral health, etc.).

## 5. Conclusions

When viewing the stunning correlations between various neurodegenerative diseases and oral biofilms, it seems attractive to link the two phenomena together in some way. However, given the current evidence, it seems too premature to conclude such a link, at least in a causal way. However, it seems that the role of oral dysbiosis in promoting systemic inflammation may play a crucial role in several of these neurodegenerative diseases.

Oral dysbiosis has also been purported to be linked with other diseases apart from those of the brain, including cancers [96], bacterial endocarditis [9,96,97,98], aspiration pneumonia [99], diabetes [100], noma [101], cardiovascular disease [102], and adverse outcomes with pregnancies [103,104]. As many of these diseases have an inflammatory connection, it is possible that there may be common pro-inflammatory pathways involved with these diseases as well as with neurodegenerative diseases. Finding these common pathways, if there are any, has the potential to uncover crucial players in several diseases, which may drastically change the methods of their prevention and treatment. Understanding the mechanisms for which the oral microbiome can positively or negatively impact inflammation at a genus/species level is needed. The implementation of long-read sequencing technologies coupled with chromosol mapping may help understand the epigenetic impact the oral microbiome has on human disease [105,106]. This illuminates the need for more research in this area to find these common pathways and to conclude if there is a reason to causally link oral microbiota with all these diseases.

The amount of evidence supporting the connection between neurodegenerative diseases and oral dysbiosis is still very low; thus, the results of the studies related to this connection should be cautiously interpreted [107]. It seems too premature to conclude that oral biofilms “cause” various diseases—while it may be tempting to include this, it seems unlikely that the health of two completely different systems in the body directly determines the state of each other. It seems more likely that these two systems are involved in similar inflammatory pathways, which may thus play a role in increasing susceptibility of one another.

Despite these criticisms, there may still be value in noting that multiple neurodegenerative diseases are similarly correlated to oral dysbiosis, particularly with periodontitis. These data may potentially suggest that there may be some kind of neuropathological pathway between periodontitis and neurodegenerative processes that are not yet understood [90]. Nonetheless, there is more research to be performed to establish whether this connection is valid. More animal studies are necessary to strengthen these connections and establish causal roles.

## Data Availability

Not applicable.
